# Altered Expressions of NF1 and NF1-Related microRNAs as Biomarkers in the Diagnosis of Undifferentiated Pleomorphic Sarcoma

**DOI:** 10.3389/fgene.2022.870191

**Published:** 2022-04-26

**Authors:** Peng Zhang, Lingling Huang, Pengwei Ma, Xiaoying Niu

**Affiliations:** ^1^ Department of Bone and Soft Tissue Cancer, The Affiliated Cancer Hospital of Zhengzhou University (Henan Cancer Hospital), Zhengzhou, China; ^2^ Department of Toxicology, College of Public Health, Zhengzhou University, Zhengzhou, China

**Keywords:** soft tissue sarcomas, undifferentiated pleomorphic sarcoma, NF1, microRNA, biomarkers

## Abstract

**Objective:** Undifferentiated pleomorphic sarcoma (UPS) is a highly malignant, aggressive, and pleomorphic subtype of soft tissue sarcoma in adults. However, UPS is difficult to be diagnosed due to the lack of specific morphological and immunophenotypic features. Here, we aimed to identify new biomarkers for the diagnosis of UPS.

**Methods:** The mRNA and protein expression of neurofibromin 1 (NF1) in 68 pairs of UPS and adjacent normal tissues were detected by qRT-PCR and immunohistochemistry, and the correlation between the NF1 protein expression and clinicopathological characteristics was analyzed. Then, differentially expressed microRNAs (DE miRNAs) were identified between the UPS tumor tissue and matched adjacent normal tissue using Hisep sequencing, Gene Ontology (GO), and Kyoto Encyclopedia of Genes and Genomes (KEGG). The DE miRNAs of the regulating NF1 gene were also identified using the TargetScan and miRanda databases and validated by qRT-PCR.

**Results:** Compared with the adjacent normal tissue, both mRNA and protein expressions of NF1 in the UPS tumor tissue were significantly decreased, and the positive rate of NF1 protein was associated with the tumor size, metastasis, and recurrence. A total of 125 known DE miRNAs were identified from the screened miRNAs based on | log_2_(Fold Change) ≥5 and *p*-value < 0.05 (A total of 82 upregulated and 43 downregulated DE miRNAs in the UPS tissue). Target genes regulated by the DE miRNAs were enriched in pathways of metabolisms, RNA degradation, PI3K-Akt, and Rap1 pathway. In total, 11 miRNAs which were predicted to regulate the *NF1* gene were screened. After verification, the relative expressions of hsa-miR-199a-3p and hsa-miR-34a-5p were increased and decreased in the UPS tumor tissue compared with those in the adjacent normal tissue, respectively.

**Conclusion:** NF1 and NF1-related microRNAs including hsa-miR-199a-3p and hsa-miR-34a-5p may be novel biomarkers in the diagnosis of undifferentiated pleomorphic sarcoma (UPS).

## Introduction

Soft tissue sarcomas (STSs) are rare malignant tumors of mesenchymal origin with high aggressiveness and heterogeneity, including more than 50 histological subtypes. Incidence of STSs accounts for 1% in adult malignant tumors (Gamboa et al., 2020). The incidence rate of STSs is low, but they are malignant and easy for metastasis and reoccurrence. Undifferentiated pleomorphic sarcoma (UPS) is a highly malignant and pleomorphic subtype of STSs in adults (Winchester et al., 2018), accounting for more than 20% of STSs, which is the highest than the other subtypes of STSs (Ray-Coquard et al., 2018). However, UPS is considered an exclusionary diagnosis due to the lack of specific morphological and immunophenotypic features (Roland et al., 2016), and its molecular changes have not yet been clarified.

Genetic alterations are always associated with oncogenesis. Neurofibromin 1 (NF1) is derived from neurofibromatosis type I, which is an autosomal dominant genetic disease with the mutation of the *NF1* gene. In addition, *NF1* is a tumor suppressor gene; NF1 mutation was first demonstrated in the malignant peripheral nerve sheath tumor (MPNST), which is a subtype of STSs (Amirnasr et al., 2020). In addition to that, its mutations were found in other subtypes of STSs. For example, there was the deletion of the *NF1* gene in pediatric rhabdomyosarcoma (Walther et al., 2016); 20% cases with mutation in the *NF1* gene were observed in 86 liposarcomas using the whole-exome sequencing (Kanojia et al., 2015). In a mouse study, the mice with NF1 deletion developed either high-grade myogenic sarcomas or MPNSTs (Dodd et al., 2013), and in our previous study, we found that individuals carrying the TC/CC genotype for NF1 rs2905789 may be susceptible to STSs (Zhang et al., 2021). Since UPS is a main kind of myogenic sarcomas and the constituent ratio of UPS is the highest among the subtypes of STSs, NF1 may be involved in the development of UPS. NF1 protein is a tumor suppressor protein; impaired NF1 may increase the Ras activity and then activate the PI3K-Akt signal pathway, which regulates tumor cell growth, survival, and angiogenesis. However, there is no report on the expression of NF1 in UPS.

MicroRNAs (miR or miRNAs) are a class of single-stranded 19–25 ribonucleotide noncoding RNAs (Saliminejad et al., 2019). They are involved in regulating the expression of about 60% of the coding genes and are widely involved in almost all the physiological processes, such as cell differentiation, proliferation, and apoptosis (Wang J. et al., 2021). In recent years, many studies have found that miRNAs are abnormally expressed in the tumor tissue and have confirmed that miRNAs are closely related to tumorigenesis (Li et al., 2020, Tang et al., 2021). Previous studies have demonstrated that antioncogene *NF1* may be regulated by some miRNAs during the occurrence of lung squamous cell carcinoma (Guo et al., 2020), ovarian cancer (Su et al., 2019), and melanoma (Stark et al., 2015), but the miRNA profile in the UPS sarcoma tissue and miRNAs, which regulate NF1 in the initiation of UPS, are still unknown.

In this study, we first investigated the expression level, clinical significance, and signal pathway of NF1 in the UPS sarcoma tissue; then, we identified the miRNA profile between the UPS tumor tissue and adjacent normal tissue and screened the miRNAs that may regulate the *NF1* gene that are expressed differentially. More understanding of molecular changes involved in UPS may provide more clues and ideas of identifying biomarkers for the diagnosis and treatment of UPS patients.

## Materials and Methods

### Subjects and Sample Collection

A total of 68 patients with primary UPS without radiotherapy and chemotherapy before surgery at the Department of Bone and Soft Tissue Sarcoma, Henan Cancer Hospital in China from 2006 to December 2016 were chosen. Among the patients, there were 39 males and 29 females, aged 55.58 ± 3.94 years.

The paired UPS tissue and normal muscle tissue which are more than 5 cm away from the sarcoma were surgically obtained, which were stored for immunohistochemistry and RNA isolation. All the patients were surveyed by a questionnaire about the basic information and signed the informed consent, and the study was approved by the Medical Ethics Committee of Henan Cancer Hospital.

### Immunohistochemistry

The protein expression of NF1, as well as the phosphorylated proteins of p-Akt, p-mTOR, and p-S6 in the adjacent normal tissue and UPS sarcoma tissue of 68 patients, was assessed by immunohistochemistry (IHC). Briefly, the specimens were deparaffinized, blocked with goat serum for 30 min, and incubated with the rabbit antihuman NF1 antibody (1: 100, Beijing Boosen Biotechnology Co., Ltd., China) at 4°C overnight; then, they were incubated with biotinylated goat antirabbit immunoglobulin at a concentration of 1:100 at 37°C for 30 min. All the immunohistochemical-stained tissue sections were assessed independently by two pathologists in a blinded manner, and a consensus was reached for each score. If there was a disagreement between these two pathologists, a third pathologist would be invited, and the three pathologists would come to the final conclusion. Scoring was based on the percentage of the positively stained cells (0=<5%; 1 = 6–25%; 1 = 6–25%; 2 = 26–50%; 3 = 51–75%; and 4 = 76–100%) under five high-power vision fields, and the intensity of staining was graded as negative (score 0), weak (score 1), moderate (score 2), or strong (score 3). The final staining scores were calculated as percentage × staining intensity. Therefore, the final scores were score 0 = value 0–1 (**negative expression**), score 1 = value 2–4 (**low expression**), and score 2 = value 5–8 and score 3 = value 9–12 (**high expression**). The ratio of the positive area to the total area for the protein expression was analyzed by ImageJ software. NF1 protein was analyzed by two ranks of expression, and p-Akt, p-mTOR, and p-S6 were analyzed using the ratio of the positive area.

### RNA Extraction

The total RNA including miRNAs was isolated from approximately 100 mg of three pairs of the sarcoma tissue and adjacent normal tissue using TRIzol reagent (Invitrogen). The concentration and quality of the RNA were determined by using a NanoDrop Spectrophotometer (NanoDrop Technologies, Thermo Fisher, United States). The individual aliquots of RNA from the UPS tissue or adjacent normal tissue were pooled for Hisep deep sequencing. The extracted RNA solution of 68 pairs of tissues is stored at −80°C for the quantitative real-time PCR (qRT-PCR).

### Hisep Sequencing

The RNA pools consisted of the same amounts of total RNA from the UPS tissue and matched adjacent normal tissue, and the total RNAs were used for Hisep deep sequencing. First, the total RNA was separated by polyacrylamide gel electrophoresis, and the small RNA regions of 18–30 nucleotides were excised. Then, 5′-adapter and 3′-adapter were ligated to the small RNAs, and the small RNA-adapter molecules were reverse transcribed and amplified. Finally, the two miRNA libraries were constructed and Hisep sequenced with Illumina Hiseq 2500 (Illumina, Inc., United States). Compared with the adjacent normal tissue, a limma test was used to identify the differentially expressed miRNAs in the UPS tissue using the DEGseq R language package. Finally, the dysregulated miRNAs were chosen based on | log_2_(Fold Change) | ≥2 and *p*-value < 0.05.

### GO and KEGG Analysis

Using the databases of TargetScan and miRanda and taking the intersection, we predicted the target genes of the differentially expressed miRNAs. The target genes were annotated from four aspects of functional items, such as the biological process, molecular function, cellular component, and signaling pathway using the databases of the Gene Ontology (GO) and Kyoto Encyclopedia of Genes and Genomes (KEGG). The differentially expressed miRNAs of the regulating *NF1* gene were identified using the TargetScan and miRanda databases.

### qRT-PCR

The miDETECT A Track™ miRNA qRT-PCR Starter Kit (Ribobio Biotechnology Co., Guangzhou, China) was used for miRNA detection. First, poly (A) tails were added to RNAs, and the poly (A) tailing reaction system was made as follows: 1.0 µL total RNA, 2.0 µL 5×poly (A) polymerase buffer, 1.0 µL poly (A) polymerase, and RNase-free water were added to 10.0 µL. The mixture was kept at 37°C for 1 h. Second, cDNA was synthesized with specific primers. We made the reverse transcription reaction system as follows: 4.0 µL RTase mix, 2.0 µL mi*DETECT* A Track™ Uni-Reverse Primer, and 10.0 µL tail product. The reaction mixtures were incubated at 42°C for 1 h, at 72°C for 10 min, and then stored at 4°C. The amplification reaction was performed in a 20 µL volume containing 2.0 µL cDNA, 0.5 µL mi*DETECT* A Track™ miRNA-Forward Primer and Reverse Primer, respectively (Ribobio Biotechnology Co., Guangzhou, China), 10.0 µL of 2×SYBR Green Mix and 7.0 µL RNase-free water. qPCR was performed as follows: 95°C for 10 min, followed by 40 cycles of 95°C for 2 s, 60°C for 20 s, and 70°C for 10 s. U6 snRNA was used as a control for the normalization of tissue miRNA levels. The primer sequences are as follows: for human NF1: forward 5′-TGG​GAC​ATT​CGC​CTC​TTA​AC-3′ and reverse 5′-ACA​CAT​GCA​AAA​TGG​GAA​CA-3’; human GAPDH: forward 5′-GGA​AGC​TTG​TCA​TCA​ATG​GAA​ATC-3′ and reverse 5′-TGA​TGA​CCC​TTT​TGG​CTC​CC-3’.

The relative expression levels of miRNAs and NF1 mRNA were calculated using a comparative Ct (2^−∆∆Ct^) method.

### Statistical Analysis

The SPSS21.0 software package was used for the data statistical analysis. The histogram was drawn with GraphPad Prism 5. The continuity variable was expressed as the mean ± SD, and the quantitative data were analyzed using two independent samples. Student’s t-test and the Pearson’s Chi-squared (x^2^) test was used to evaluate the positive rates of NF1 between the UPS sarcoma tissue and adjacent normal tissue and the difference between the protein levels of NF1 staining in the UPS sarcoma tissue and clinicopathological characteristics, respectively. The *p* value less than 0.05 was considered statistically significant.

## Results

### The Expression of NF1 in the UPS Sarcoma Tissue and Adjacent Normal Tissue

**FIGURE 1:**
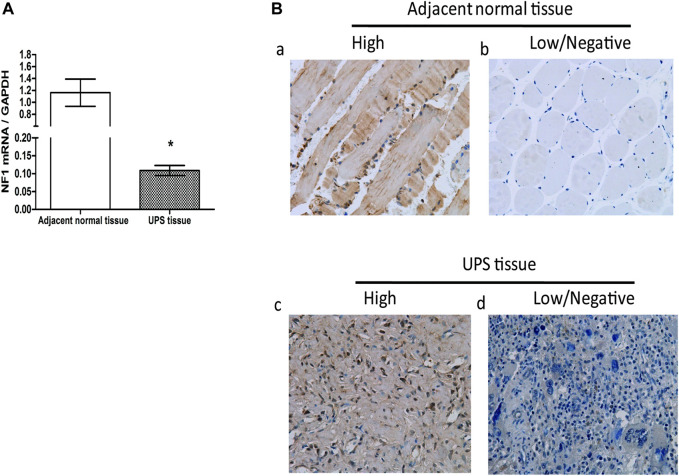
Expression of NF1 in the UPS sarcoma tissue and adjacent normal tissue. **(A)**: NF1 mRNA expression in the UPS sarcoma tissue and adjacent normal tissue by qRT-PCR, *: vs. adjacent normal tissue, *p* < 0.05; **(B)** Representative images of the protein expression of NF1 in the UPS sarcoma tissue and adjacent normal tissue by immunohistochemistry (200×) (a): High protein expression of NF1 in the adjacent normal tissue; (b): Low or negative protein expression of NF1 in the adjacent normal tissue; (c): High protein expression of NF1 in the UPS sarcoma tissue; (d): Low or negative protein expression of NF1 in the UPS sarcoma tissue.

Figure 1A demonstrated that the NF1 mRNA level in the UPS tissue was decreased compared to that in the adjacent normal tissue, and the difference was statistically significant (*p* < 0.05). NF1 protein was stained mainly in the nucleus, and a high expression of protein was considered as a positive expression. In the 68 cases of the adjacent normal tissue, there were 48 cases with NF1 high protein expression (Figure 1B a) and 20 cases with NF1 low/ no protein expression (Figure 1B b); In the 68 cases of the UPS sarcoma tissue, there were 27 cases with NF1 high protein expression (Figure 1B c) and 41 cases with NF1 low/no protein expression (Figure 1B d). In the adjacent normal tissue, the positive rate of NF1 protein was 70.59%, and in the UPS tissue, the positive rate of NF1 protein was 39.71%. The positive rate of NF1 protein in the UPS sarcoma tissue was decreased compared to that in the adjacent tissue, and the difference was statistically significant (*p* < 0.05) (Table 1).

**TABLE 1:** Protein expression of NF1 in the UPS tissue and adjacent normal tissue.

Group	N	NF1 (n, %)	
		High	Low/Negative
Adjacent normal tissue	68	48(70.59)	20 (29.41)
UPS tissue	68	27(39.71)	41 (60.29)
$\chi^{2}$		13.110	
*P*		**0.001**	

Bold values represents p < 0.05.

### Correlation Between the NF1 Protein Expression and Clinicopathological Characteristics of UPS Patients

We divided the high expression and low/negative expression groups based on the protein expression of NF1 and assessed the correlation between the NF1 protein expression and clinicopathological characteristics of the UPS patients. The results (Table 2) showed that the NF1 protein expression was associated with the tumor size (x^2^ = 7.372 and *p* = 0.007), distant metastasis (x^2^ = 9.378 and *p* = 0.002), and recurrence (x^2^ = 4.300 and *p* = 0.047), but NF1 was not associated with gender, age, lymph node metastasis, location, or 5-year survival.

**TABLE 2:** Correlation between the NF1 protein expression and clinicopathological characteristics of UPS patients

Characteristic	N	NF1 protein expression		x^2^	*p*
		High (*n* = 27)	Low/Negative (*n* = 41)		
Gender					
Male	39	12	27	3.051	0.081
Female	29	15	14		
Age (years)					
<55	24	11	13	0.582	0.446
≥55	44	16	28		
Tumor size (cm)					
<5	16	11	5	7.372	**0.007**
≥5	52	16	36		
Lymph node metastasis					
Yes	23	7	16	1.248	0.264
No	45	20	25		
Distant metastasis					
Yes	19	2	17	9.378	**0.002**
No	49	27	41		
Location					
Trunk	32	12	20	0.123	0.726
Extremity	36	15	21		
Recurrence					
Yes	28	7	21	4.300	**0.047**
No	40	20	20		
5-year survival					
Survival	45	20	25	1.248	0.305
Dead	23	7	16		

Bold values represents p < 0.05.

### The Protein Levels of p-Akt, p-mTOR, and p-S6 Were Increased in the UPS Sarcoma Tissue

**FIGURE 2:**
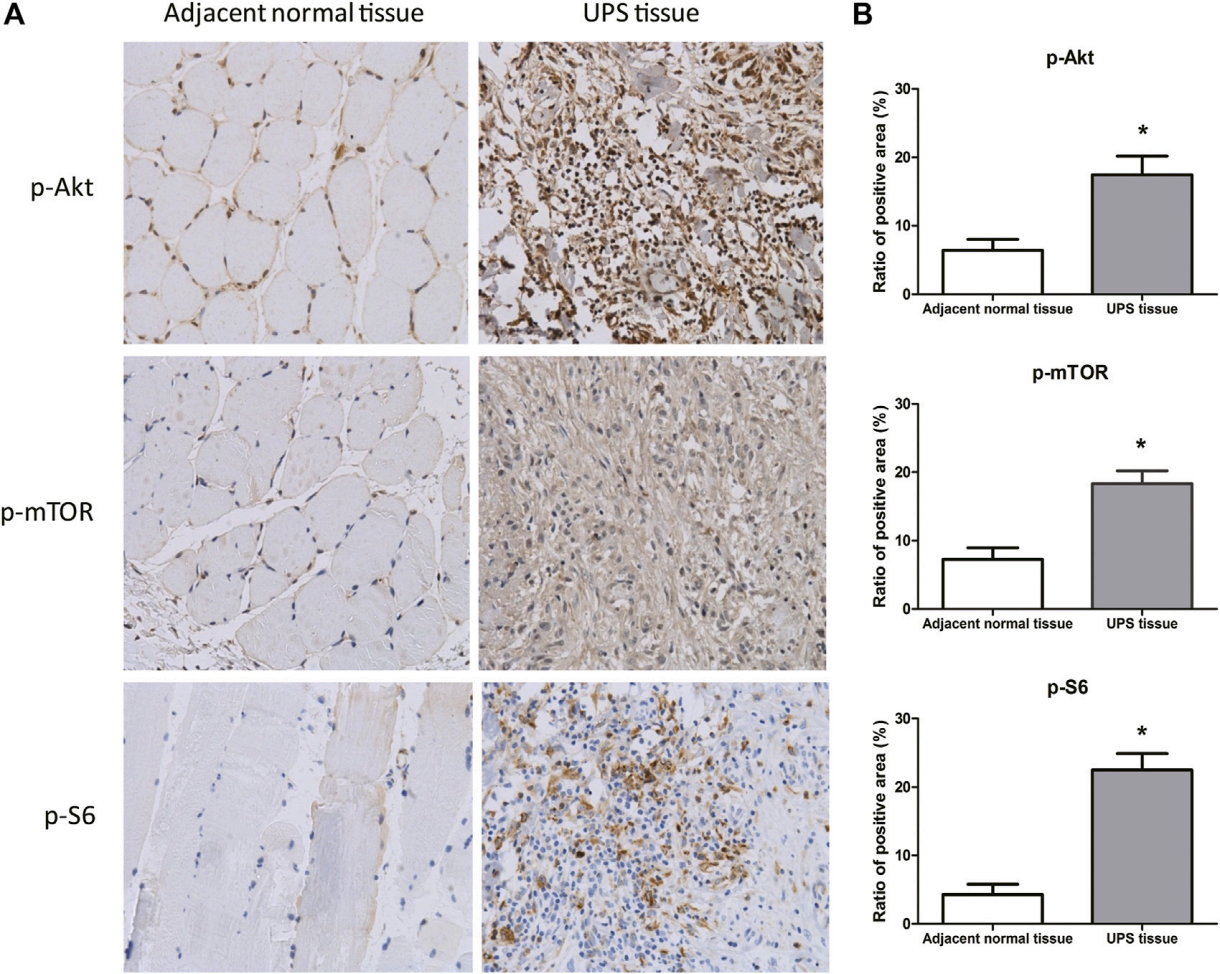
Protein expression of p-Akt, p-mTOR, and p-S6 in the UPS sarcoma tissue and adjacent normal tissue. **(A)**: Representative images of the protein expression of p-Akt, p-mTOR, and p-S6 in the UPS sarcoma tissue and adjacent normal tissue by immunohistochemistry (200×); **(B)**: The comparison of the ratios of the positive area to the total area of p-Akt, p-mTOR, and p-S6 between the UPS sarcoma tissue and adjacent normal tissue. *: vs. adjacent normal tissue, *p* < 0.05.

The phosphorylated protein of p-Akt, p-mTOR, and p-S6 in the adjacent normal tissue and UPS sarcoma tissue from the 68 patients were detected by immunohistochemistry (Figure 2A). p-Akt and p-mTOR were stained brown color in both the cytoplasm and nucleus, and p-S6 was stained in the cytoplasm. The ratios of the positive area to the total area of p-Akt, p-mTOR, and p-S6 determined by ImageJ software were (17.44 ± 2.73)%, (18.32 ± 1.90)%, and (22.52 ± 2.39)%, which were significantly increased compared to those in the adjacent tissue ((6.41 ± 1.60)%, (7.25 ± 1.74)%, and (4.29 ± 1.50)%, respectively) (*p* < 0.05) (Figure 2B).

### Hisep Sequencing of Noncoding RNA in the Tissue

Hisep sequencing yielded a total of 22,273,789 and 22,506,782 raw sequence reads in the adjacent normal tissue and UPS tissue, respectively. The types and numbers of the small RNAs (sRNAs) mapped to the genome by SOAP and bowtie are shown in Supplementary Table S1 and Supplementary Figures S1A, B. Supplementary Figure S1C demonstrated that the size distribution of the reads indicated that the majority of sRNAs were 22 nt in the tissue. The percentages of miRNAs in total sRNAs in the adjacent normal tissue and UPS tissue were 67.76 and 77.97%, respectively (Supplementary Table S1), which indicated that the sRNAs were highly enriched in the miRNA sequences.

### Analysis of Differentially Expressed miRNAs

**FIGURE 3:**
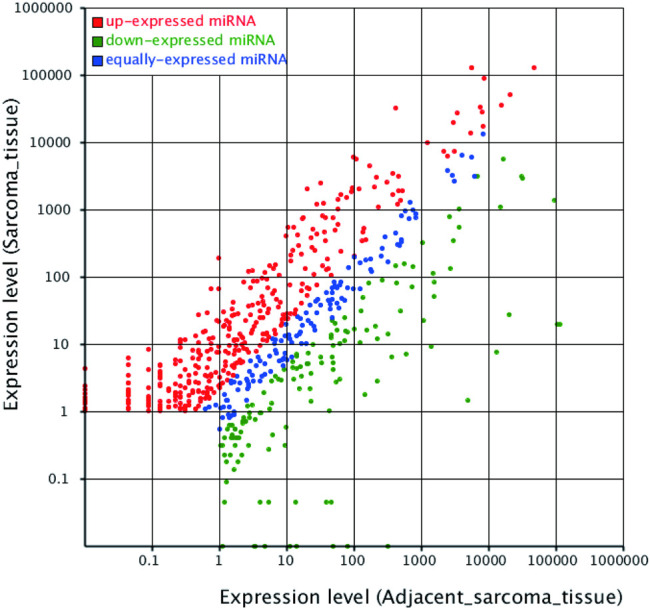
Scatter plot indicating the differentially expressed miRNAs between the adjacent normal tissue and UPS tissue. The principles of identifying the differentially expressed miRNAs were based on | log_2_(Fold Change) | ≥2 and *p*-value <0.05. Each dot represented one miRNA. The red dots represented upregulated miRNAs, the green dots represented downregulated miRNAs, and the blue dots represented equally-expressed miRNAs.

A total of 496 known differentially expressed miRNAs were identified from the screened miRNAs with Hisep sequencing based on | log_2_(Fold Change) | ≥2 and *p*-value < 0.05. The scatter plot demonstrated in Figure 3 showed that there were many dysregulated miRNAs between the adjacent normal tissue and UPS tissue. In order to pick up more important miRNAs with a differential expression, more restrictive criteria were used as follows: more than 50 copies, | log_2_ (Fold Change) | ≥5 and *p*-value < 0.05. According to these principles, 125 differentially expressed known miRNAs were identified, of which 82 were upregulated (Supplementary Table S2) and 43 downregulated (Supplementary Table S3) in the UPS tissue. Top 10 of the upregulated and downregulated differentially expressed known miRNAs are shown in Table 3.

**TABLE 3:** Top 10 upregulated and downregulated differentially expressed known miRNAs in the UPS tumor tissue compared with the adjacent normal tissue.

miRNA ID	Copy number in the adjacent normal tissue	Normalized miRNA level in the adjacent normal tissue	Copy number in the UPS tissue	Normalized miRNA level in the UPS tissue	Fold change	*p* value
hsa-miR-138-5p	460	20.44	44,805	2,011.56	98.42	0
hsa-miR-376a-3p	725	32.21	55,637	2,497.87	77.54	0
hsa-miR-155-5p	2,233	99.21	135,439	6,080.64	61.29	0
hsa-miR-146a-5p	2,421	107.57	125,240	5,622.75	52.27	0
hsa-miR-409-3p	240	10.66	12,023	539.78	50.62	0
hsa-miR-299-3p	61	2.71	2,713	121.80	44.94	0
hsa-miR-31-5p	630	27.99	26,972	1,210.93	43.26	0
hsa-miR-146b-5p	397	17.64	16,985	762.56	43.23	0
hsa-miR-21-3p	392	17.42	16,359	734.45	42.17	0
hsa-miR-214-5p	289	12.84	12,059	541.40	42.16	0
hsa-miR-133a-3p	2,579,106	114,592	438	20	−5,827.40	0
hsa-miR-1-3p	2,398,804	106,581	440	20	−5,395.38	0
hsa-miR-206	295,147	13,114	171	8	−1,708.13	0
hsa-miR-499a-5p	459,811	20,430	612	27	−743.55	0
hsa-miR-95-3p	31,853	1,415	203	9	−155.29	0
hsa-miR-378a-5p	13,374	594	159	7	−83.24	0
hsa-miR-378d	4,958	220	62	3	−79.14	0
hsa-miR-378a-3p	2,171,985	96,504	30,941	1,389	−69.47	0
hsa-miR-486-5p	8,200	364	144	6	−56.35	0
hsa-miR-34a-5p	24,240	1,077	492	22	−48.76	0

### The Analysis of Gene Ontology Enrichment and Kyoto Encyclopedia of Genes and Genomes Pathways With Target Genes Regulated by DE miRNAs

**FIGURE 4:**
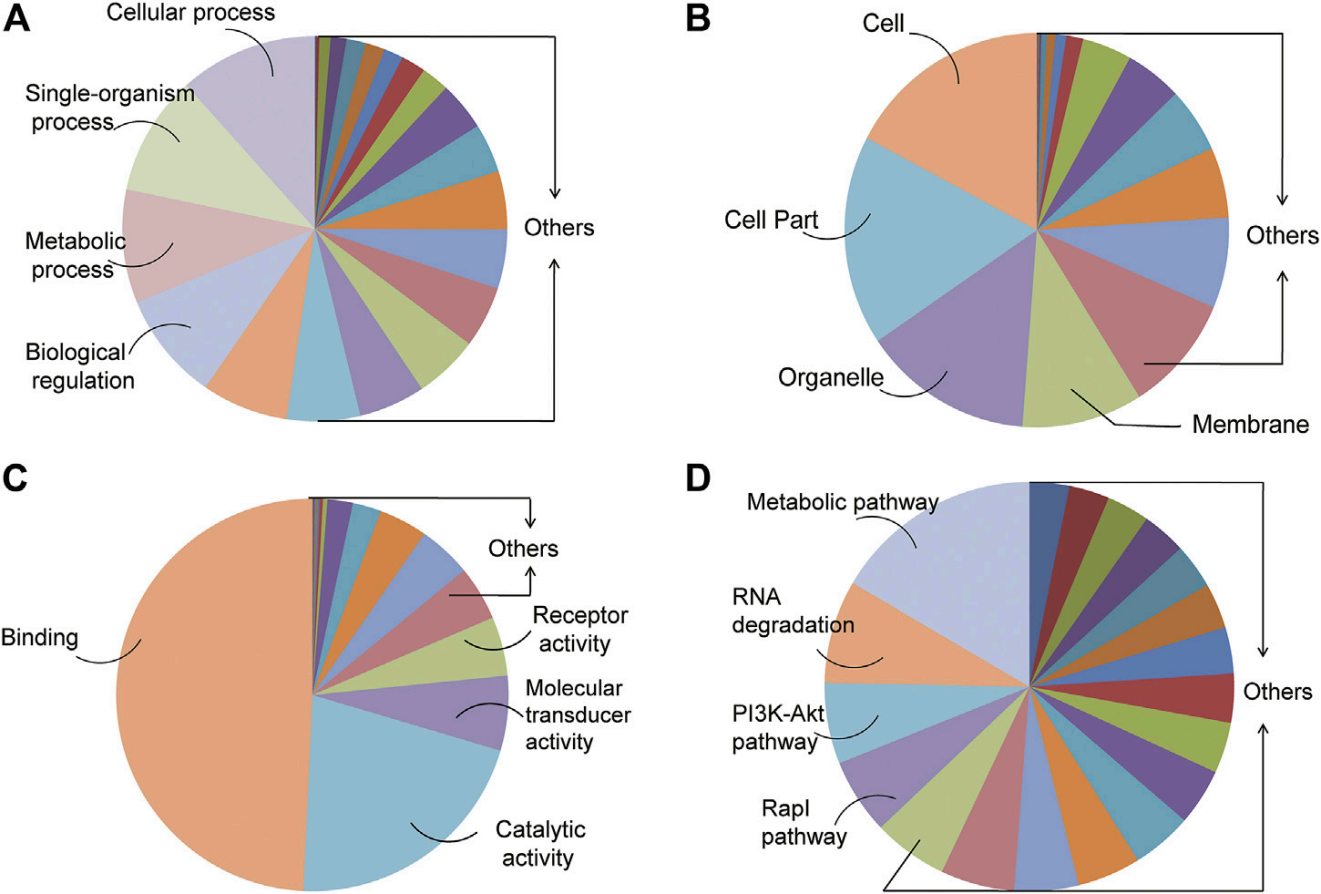
GO and KEGG analysis of genes targeted by the DE miRNAs. **(A–C)** Top four GO biological processes (BPs), cellular components (CCs), and molecular functions (MFs) enriched in target genes regulated by all the DE miRNAs. **(D)** KEGG enrichment analysis of signal pathways.

A total of 51,449 target genes which may be regulated by 496 DE miRNAs were identified from the intersection of TargetScan and miRanda databases. In order to investigate the possible mechanism for the initiation of UPS, we conducted the analysis of GO enrichment including the biological process (BP), cellular component (CC), molecular function (MF), and KEGG signal pathway. The BP demonstrated the physical activities regulated by the target genes associated with DE miRNAs, which were presented with the cellular process, single-organism process, metabolic process, and biological regulation (Figure 4A). The CC showed the location of transcription and protein translation of the target genes. The cell, cell part, organelle, and membrane were in the CC category (Figure 4B). The MF defined the role of the target genes regulated by DE miRNAs. The target genes were predicted to regulate binding, catalytic activity, molecular transducer activity, and receptor activity (Figure 4C). These target genes were also enriched in some important signal pathways, such as the metabolic pathway, RNA degradation, PI3K-Akt pathway, and Rap1 pathway (Figure 4D).

### DE miRNAs Regulating NF1 Were Predicted and Verified

To screen the miRNAs which were differentially expressed and predicted to regulate the gene of NF1 from 125 DE known miRNAs (log_2_ (Fold Change) | ≥5 and *p*-value < 0.05), first, the miRNAs that may regulate NF1 were chosen from the databases of TargetScan and miRanda and then were intersected with the screened 125 DE known miRNAs; finally, 11 miRNAs were screened, which are demonstrated in Table 4.

**TABLE 4:** Eleven DE miRNAs regulated the *NF1* gene were predicted using bioinformatics.

Name of the regulated gene	miRNA name	Fold change	*p*	Expression level in the tumor tissue
NF1	**hsa-miR-127-3p**	26.03	0	up
NF1	hsa-miR-654-5p	23.96	0	up
NF1	**hsa-miR-199a-3p**	22.33	0	up
NF1	hsa-miR-199b-3p	22.33	0	up
NF1	hsa-miR-7-5p	20.15	0	up
NF1	hsa-miR-942-5p	8.21	6.67E-147	up
NF1	**hsa-miR-34a-5p**	−48.76	0	down
NF1	hsa-miR-196-5p	−30.30	0	down
NF1	hsa-miR-144-3p	−20.18	3.93E-285	down
NF1	hsa-miR-193-3p	−20.21	0	down
NF1	hsa-miR-24-3p	−13.61	0	down

Bold values represents that the three NF1- related microRNAs were chosen and verified.

**FIGURE 5:**
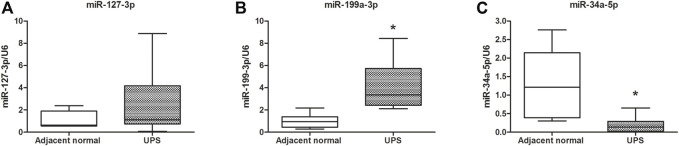
Validation by qRT-PCR of the three miRNAs regulating the *NF1* gene. **(A–C)** were the expression and comparison of miR-127-3p, miR-199a-3p, and miR-34a-5p in 68 pairs of the UPS tumor tissue and adjacent normal tissue. U6 was used as the reference gene. *: vs. adjacent normal tissue, *p* < 0.05.

Based on the literature studies and fold change values of the differential expression, hsa-miR-127-3p and hsa-miR-199a-3p, which were the first and the third of upregulation, and hsa-miR-34a-5p, which was the first of the downregulation in the UPS tissue, were selected to be verified in the 68 pairs of the UPS tissue and adjacent normal tissue. Figure 5 illustrated that the relative expression of the human miR-199a-3p and miR-34a-5p were increased and decreased in the UPS tumor tissue compared with those in the adjacent normal tissue, respectively, and the difference was statistically significant (*p <* 0.05). In addition, there was an upward trend of the level of human miR-127-3p in the UPS tissue compared to that in the adjacent normal tissue, but there was no significant difference (*p >* 0.05).

## Discussion

The *NF1* gene is mapped to human chromosome 17 and encodes neurofibromin. Point mutations and genomic deletions of the *NF1* gene were found in 10.5% of the myxofibrosarcomas and 8% of the pleomorphic liposarcomas in 207 samples of STSs (Barretina et al., 2010). However, the NF1 expression in UPS has not been explored. In this study, we detected the mRNA and protein expression of NF1 in the UPS sarcoma tissue and adjacent normal tissue and analyzed the clinicopathological characteristics. Our data demonstrated that not only NF1 mRNA was decreased but also the positive rate of NF1 protein in the UPS tissue (39.71%) was significantly decreased compared with that in the adjacent normal tissue (70.59%). A research reported that 58.1% (93/160) of the gastric cancer samples were NF1-positive as compared to 94.4% (151/160) of the matched normal tissue samples (*p* < 0.001) (Liu et al., 2017), which was consistent with our results. In addition, the NF1 protein expression was associated with the tumor size and distant metastasis of UPS in the present study. In the gastric cancer study, the NF1 protein was associated with the T stage and TNM stage (18). Actually, the T stage means the size or direct extent of the primary tumor, equal to the tumor size. Therefore, our data were consistent with the results of gastric cancer. But NF1 was not associated with gender, age, or lymph node metastasis in UPS. Elzagheid et al. demonstrated that the NF expression showed a borderline correlation with gender; tumors of women showed a higher NF expression than those of males, but there was no significant difference (*p* = 0.068) (Elzagheid et al., 2016). Further study on the expression of NF1 in UPS is of significance to understand the pathogenesis of UPS.

miRNA expression deregulation has been found in many types of cancers (Xu et al., 2014; Komoll et al., 2021), and some reports have indicated that the differentially expressed miRNAs may be employed for the diagnosis of sarcomas (Fricke et al., 2015; Zhou et al., 2016; Smolle et al., 2017). Pazzaglia et al. (2017) performed 12 miRNA expression analyses between 59 primary STS samples (27 leiomyosarcomas (LMS) and 32 UPS) and 10 normal control tissue using the TaqMan microRNA array and found that compared with the normal control tissue, there was a statistical difference only on the increasing expression of miR-152 in STS samples, and the researchers from Finland conducted miRNA profiling on 10 LMS and 10 UPS samples; two cultured human mesenchymal stem cell samples were used as controls. The results of the miRNA microarray demonstrated that miR-320a and miR-199b-5p were differentially expressed between LMS and UPS, and more samples were detected to confirm the microarray data using qRT-PCR (Guled et al., 2014). This study was carried out to look for the differentially expressed miRNAs for the diagnosis between LMS and UPS. While Pazzaglia et al. took LMS and UPS together as soft tissue sarcoma, the differentially expressed miRNAs in STSs may be less compared to normal control because the miRNA profiles are different in each subtype of STSs. However, in this study, we detected and identified the miRNA profile using miRNA sequencing between the UPS tumor tissue and adjacent normal tissue for the first time. Our data showed that 125 differentially expressed miRNAs were identified in the UPS tissue, according to | log_2_(Fold Change) | ≥5 and *p*-value < 0.05.

GO analysis was performed to analyze the function of these target genes regulated by the DE miRNAs, which included transcriptional regulation, protein binding, metabolic regulation, and signal transduction. Furthermore, KEGG analysis found that these target genes were enriched in some important signal pathways, such as the metabolic pathway, RNA degradation, PI3K-Akt pathway, and Rap1 pathway. The PI3K/Akt signaling pathway participates in the phosphorylation of numerous molecules that control the cell growth, proliferation, metabolism, and survival. This pathway is commonly activated in cancers (Ghafouri-Fard et al., 2021), including soft tissue sarcoma (Serrano et al., 2016). The PI3K/Akt signaling pathway is related closely to the tumor suppressor gene *NF1* that could play the role by inhibiting this signaling pathway (Gutmann et al., 2017). Our data showed that the NF1 expression was decreased, but the protein expressions of p-Akt, p-mTOR, and p-S6 were significantly higher in the UPS sarcoma tissue than those in the adjacent normal tissue, which confirmed and verified that the NF1-inhibited PI3K-Akt-mTOR-S6 signal pathway was associated with the occurrence and growth of UPS. Ras-associated protein 1 (Rap1) is a member of the Ras family of small G proteins that regulate a variety of signaling pathways involved in proliferation, differentiation, polarity, and apoptosis (Zhang et al., 2019). Studies revealed that Rap1 is not only a tumor suppressor gene but also a conditioned oncoprotein (Hattori and Minato, 2003). An increased and abnormal activation of Rap1 can lead to tumor formation and malignant development (Shah et al., 2019). The pathways need to be explored further.

miRNAs usually play their function by negatively regulating the gene expression (Lee et al., 2016). When the target gene of miRNA is an oncogene, the decreased expression of miRNA could cause the upregulation of oncogene, which will eventually lead to excessive cell proliferation, reduced apoptosis, and tumor formation. When the target genes of miRNAs are tumor suppressor genes, the miRNA function of oncogenes and the increased expression of these miRNAs can cause downregulation of their target genes (tumor suppressor genes) and promote tumorigenesis. In this study, 11 miRNAs were screened, which were predicted to regulate the gene of NF1 from 125 DE known miRNAs. hsa-miR-127-3p, hsa-miR-199a-3p, and hsa-miR-34a-5p were validated by qRT-PCR in 68 pairs of the UPS tissue and adjacent normal tissue.

The previous literature studies have reported that the differential expression of hsa-miR-127-3p in different tumors is not consistent. For example, hsa-miR-127-3p is downregulated in oral squamous cell carcinoma and inhibits tumor proliferation and metastasis as a tumor suppressor (Ji et al., 2019). Jiang et al. found that hsa-miR-127-3p is downregulated in the glioblastoma tissue compared with the normal brain tissue through the next-generation sequencing analysis of miRNA (Jiang et al., 2014). However, the upregulation of hsa-miR-127-3p in the colorectal cancer tissue was associated with KRAS mutation (Mosakhani et al., 2012). Also, in this study, the results of miRNA sequencing demonstrated that the expression of hsa-miR-127-3p was increased in the UPS tumor tissue, but there was no statistical significance of hsa-miR-127-3p in the later verification, which needs to be validated with more samples.

Many reports have demonstrated that hsa-miR-199a-3p is a kind of oncogenic miRNAs. Zhenqiang Wang (Wang et al., 2014) found that hsa-miR-199a-3p was significantly upregulated in the gastric cancer cell lines and tissue and that hsa-miR-199a-3p dramatically increased the cell proliferation and suppressed cell apoptosis both *in vitro* and *in vivo*, which is a tumor promoter. Another study (Wan et al., 2013) reported that the hsa-miR-199a-3p expression was significantly upregulated in the colorectal cancers tissue than the normal adjacent tissue, and a high hsa-miR-199a-3p expression contributed to more advanced lymphatic invasion, lymph node metastasis, liver metastases, late TNM stage of colorectal cancer, and shorter overall survival rate. Furthermore, the hsa-miR-199a-3p inhibitor could markedly inhibit the colon cancer cell proliferation and induce more cell apoptosis. But the downregulation of hsa-miR-199a-3p was observed in the tissue of esophageal squamous cell carcinoma (Hou et al., 2021) and hepatocellular carcinoma (Giovannini et al., 2018). From the researches abovementioned, we found that the expression of hsa-miR-199a-3p is not consistent. In the present study, the hsa-miR-199a-3p expression is increased in the UPS tissue.

The relative expression and function of hsa-miR-34a-5p is similar. The expression level of hsa-miR-34a-5p is decreased in neuroblastoma, and hsa-miR-34a-5p may act as a tumor suppressor (Wang Y. et al., 2021). Ding et al. (2017) reported that the level of hsa-miR-34a-5p was downregulated in the ovarian cancer cells, and the hsa-miR-34a-5p overexpression suppressed the ovarian cancer cell proliferation and triggered apoptosis. In addition, hsa-miR-34a-5p was downregulated in the pancreatic cancer tissue, which was associated with proliferation, metastasis, and invasion of the pancreatic cancer cells (Sun et al., 2018). In this study, hsa-miR-34a-5p is downregulated in the UPS tumor tissue, which was consistent with the previous studies performed in other cancers. It inferred that hsa-miR-34a-5p might be a tumor suppressor.

**FIGURE 6:**
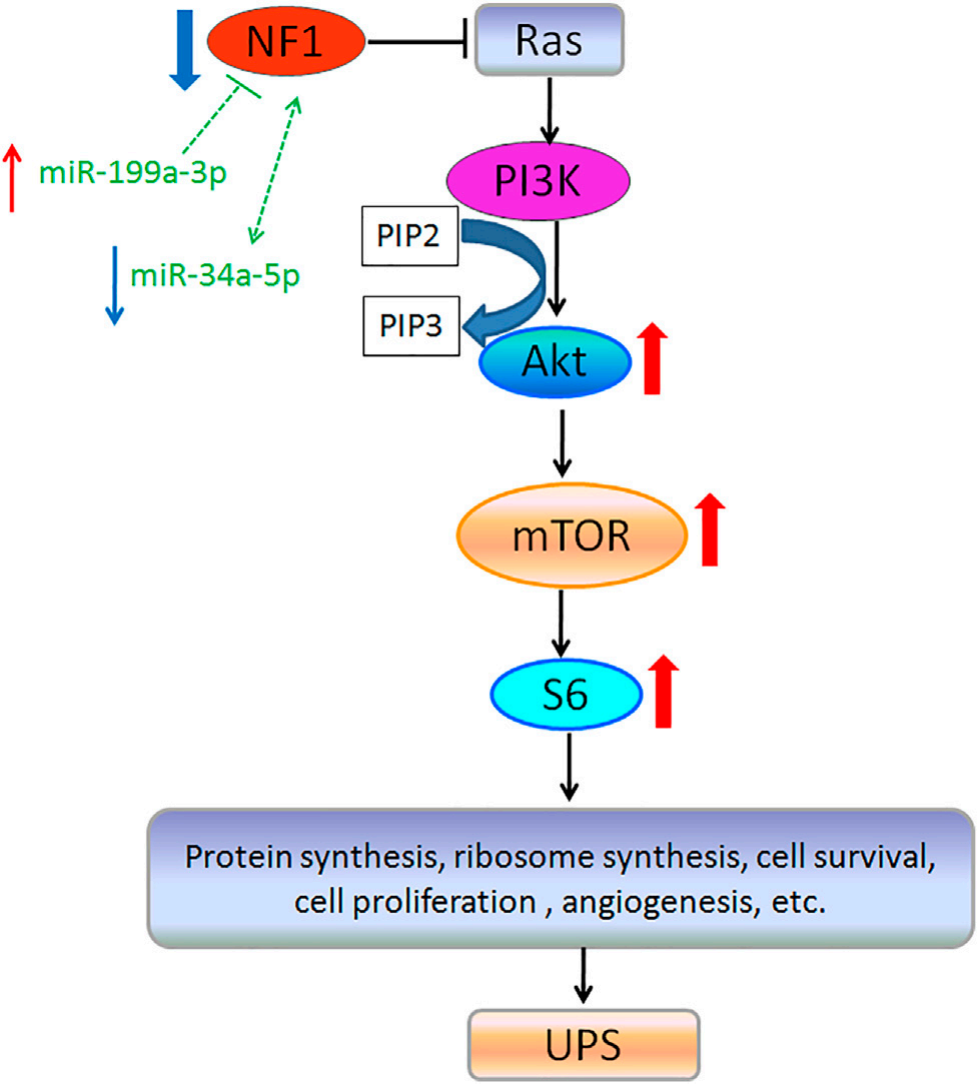
Conclusion image of NF1 and NF1-related miRNAs involved in signal pathways and occurrence of UPS. Note: Blue arrows: downregulated expression; Red arrows: upregulated expression; and Dotted line: Need to be validated.

In a word, in this study, first the mRNA and protein expression of NF1 was decreased in the UPS tumor tissue, and NF1 protein was associated with some clinical characteristic, such as tumor size, distant metastasis, and recurrence *via* the PI3K-Akt-mTOR-S6 signal pathway. Second, we identified the miRNA profile and screened 125 known differently expressed miRNAs of the UPS tumor tissue compared with the matched adjacent normal tissue using microRNA sequencing. In total, 11 miRNAs that may regulate the *NF1* gene was screened among the differently expressed miRNAs, and three miRNAs were verified; we found that hsa-miR-199a-3p was upregulated, and hsa-miR-34a-5p was downregulated (Figure 6). In this study, the altered expression of NF1 and NF1-related microRNAs, such as hsa-miR-199a-3p and hsa-miR-34a-5p may be biomarkers, which provide a basis for the novel strategy of UPS diagnosis in future.

## Data Availability

The datasets presented in this study can be found in online repositories. The names of the repository/repositories and accession number(s) can be found below: https://www.ncbi.nlm.nih.gov/bioproject/PRJNA806207, PRJNA806207.

## Supplementary Material

Supplementary Figure S1 Size distribution and annotation of small RNAs from the adjacent normal tissue and UPS tissue. The proportions of various kinds of small RNAs detected in the adjacent normal tissue **(A)** and UPS tissue **(B)**. **(C)** Frequency percent of length distribution (nt) of the annotated reads of the miRNAs from the adjacent normal tissue and UPS tissue.
