# Supplementary figures and images for: Altered Expressions of NF1 and NF1-Related microRNAs as Biomarkers in the Diagnosis of Undifferentiated Pleomorphic Sarcoma
Source: Front Genet. 2022 Apr 26;13:870191. doi: 10.3389/fgene.2022.870191 (PMC9086456; doi:10.3389/fgene.2022.870191)

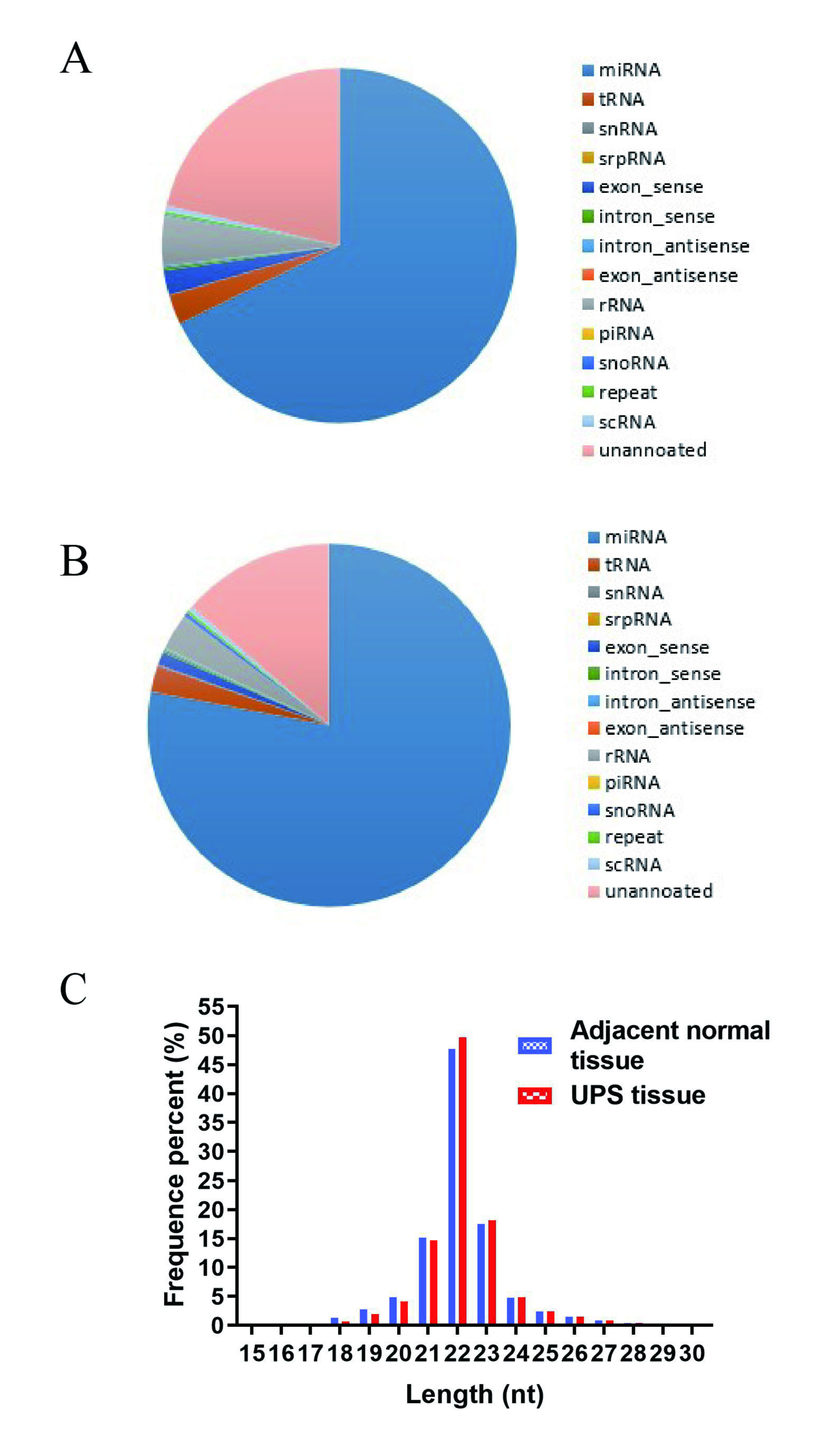

Supplement: Supplementary file 1 [file Image1.TIF]
