# Supplementary material for: Altered Expressions of NF1 and NF1-Related microRNAs as Biomarkers in the Diagnosis of Undifferentiated Pleomorphic Sarcoma
Source: Front Genet. 2022 Apr 26;13:870191. doi: 10.3389/fgene.2022.870191 (PMC9086456; doi:10.3389/fgene.2022.870191)
Supplement: Supplementary file 2 [file DataSheet1.docx]

**Supplementary tables**

Supplementary Table S1 The types and numbers of the small RNAs mapped to the genome

| Types of sRNA | | Types of sRNA in adjacent normal tissues | Number of sRNA in adjacent normal tissues | Types of sRNA in UPS tumor tissues | Number of sRNA in UPS tumor tissues |
| --- | --- | --- | --- | --- | --- |
| Total sequences | 952741 | | 22506782 | 774566 | 22273789 |
| Aligning genomic sequences | | 671274 (70.46%) | 17623055(78.30%) | 457541(59.07%) | 19166494(86.05%) |
| miRNA | | 7298 (0.77%) | 15249718(67.76%) | 9389(1.21%) | 17366915(77.97%) |
| tRNA | | 40578 (4.26%) | 609511(2.71%) | 34996(4.52%) | 522860(2.35%) |
| snRNA | | 3366(0.35%) | 11677(0.05%) | 3979(0.51%) | 28937(0.13%) |
| srpRNA | | 173(0.02%) | 481(0.002%) | 297(0.04%) | 1361(0.01%) |
| exon_sense | | 392015(41.15%) | 499221(2.22%) | 191941(24.78%) | 253031(1.14%) |
| intron_sense | | 53662(5.63%) | 58883(0.26%) | 38012(4.91%) | 46846(0.21%) |
| intron_antisense | | 10884(1.14%) | 34725(0.15%) | 24921(3.22%) | 35580(0.16%) |
| exon_antisense | | 3001(0.31%) | 5994(0.03%) | 7943(1.03%) | 12812(0.06%) |
| rRNA | | 99131(10.40%) | 1013076(4.50%) | 87097(11.24%) | 726062(3.26%) |
| piRNA | | 108(0.01%) | 714(0.003%) | 119(0.02%) | 1266(0.01%) |
| snoRNA | | 4290(0.45%) | 29153(0.13%) | 5885(0.76%) | 83930(0.38%) |
| repeat | | 53649(5.63%) | 65795(0.29%) | 37957(4.90%) | 54889(0.25%) |
| scRNA | | 1684(0.18%) | 120417(0.54%) | 1245(0.16%) | 96254(0.43%) |
| unannoated | | 282902(29.69%) | 4807417(21.36%) | 330785(42.71%) | 3043046(13.66%) |

Supplementary Table S2 82 upregulated differentially expressed known miRNAs in UPS tumor tissues compared with adjacent normal tissues

| miRNA ID | Copy number in  Adjacent normal tissue | Normalized miRNA level in Adjacent normal tissue | Copy number in UPS tissue | Normalized miRNA level in UPS tissue | Fold change | *P* value |
| --- | --- | --- | --- | --- | --- | --- |
| hsa-miR-138-5p | 460 | 20.44 | 44805 | 2011.56 | 98.42 | 0 |
| hsa-miR-376a-3p | 725 | 32.21 | 55637 | 2497.87 | 77.54 | 0 |
| hsa-miR-155-5p | 2233 | 99.21 | 135439 | 6080.64 | 61.29 | 0 |
| hsa-miR-146a-5p | 2421 | 107.57 | 125240 | 5622.75 | 52.27 | 0 |
| hsa-miR-409-3p | 240 | 10.66 | 12023 | 539.78 | 50.62 | 0 |
| hsa-miR-299-3p | 61 | 2.71 | 2713 | 121.80 | 44.94 | 0 |
| hsa-miR-31-5p | 630 | 27.99 | 26972 | 1210.93 | 43.26 | 0 |
| hsa-miR-146b-5p | 397 | 17.64 | 16985 | 762.56 | 43.23 | 0 |
| hsa-miR-21-3p | 392 | 17.42 | 16359 | 734.45 | 42.17 | 0 |
| hsa-miR-214-5p | 289 | 12.84 | 12059 | 541.40 | 42.16 | 0 |
| hsa-miR-382-5p | 220 | 9.77 | 8945 | 401.59 | 41.08 | 0 |
| hsa-miR-889-3p | 71 | 3.15 | 2718 | 122.03 | 38.68 | 0 |
| hsa-miR-31-3p | 52 | 2.31 | 1856 | 83.33 | 36.07 | 0 |
| hsa-miR-574-5p | 802 | 35.63 | 27316 | 1226.37 | 34.42 | 0 |
| hsa-miR-224-5p | 332 | 14.75 | 9397 | 421.89 | 28.60 | 0 |
| hsa-miR-99b-3p | 72 | 3.20 | 1896 | 85.12 | 26.61 | 0 |
| hsa-miR-432-5p | 126 | 5.60 | 3270 | 146.81 | 26.22 | 0 |
| hsa-miR-218-5p | 1442 | 64.07 | 37315 | 1675.29 | 26.15 | 0 |
| hsa-miR-127-3p | 3815 | 169.50 | 98289 | 4412.77 | 26.03 | 0 |
| hsa-miR-320b | 108 | 4.80 | 2571 | 115.43 | 24.05 | 0 |
| hsa-miR-654-5p | 82 | 3.64 | 1944 | 87.28 | 23.96 | 0 |
| hsa-miR-221-3p | 1318 | 58.56 | 31114 | 1396.89 | 23.85 | 0 |
| hsa-miR-342-5p | 66 | 2.93 | 1542 | 69.23 | 23.61 | 0 |
| hsa-miR-424-3p | 763 | 33.90 | 17770 | 797.80 | 23.53 | 0 |
| hsa-miR-199b-3p | 128468 | 5707.97 | 2839245 | 127470.23 | 22.33 | 0 |
| hsa-miR-199a-3p | 128480 | 5708.50 | 2839403 | 127477.32 | 22.33 | 0 |
| hsa-miR-222-3p | 2113 | 93.88 | 46380 | 2082.27 | 22.18 | 0 |
| hsa-miR-376c-5p | 154 | 6.84 | 3374 | 151.48 | 22.14 | 0 |
| hsa-miR-494-3p | 98 | 4.35 | 2144 | 96.26 | 22.11 | 0 |
| hsa-miR-376b-5p | 143 | 6.35 | 2982 | 133.88 | 21.07 | 0 |
| hsa-miR-376b-3p | 548 | 24.35 | 11368 | 510.38 | 20.96 | 0 |
| hsa-miR-7-5p | 2075 | 92.19 | 41377 | 1857.65 | 20.15 | 0 |
| hsa-miR-369-5p | 860 | 38.21 | 17034 | 764.76 | 20.01 | 0 |
| hsa-miR-450a-5p | 283 | 12.57 | 5511 | 247.42 | 19.68 | 0 |
| hsa-miR-214-3p | 1754 | 77.93 | 33723 | 1514.02 | 19.43 | 0 |
| hsa-miR-142-5p | 343 | 15.24 | 6443 | 289.26 | 18.98 | 0 |
| hsa-miR-369-3p | 258 | 11.46 | 4702 | 211.10 | 18.42 | 0 |
| hsa-miR-503-5p | 116 | 5.15 | 2050 | 92.04 | 17.86 | 0 |
| hsa-miR-379-5p | 1296 | 57.58 | 22850 | 1025.87 | 17.82 | 0 |
| hsa-miR-320a | 2662 | 118.28 | 44788 | 2010.79 | 17.00 | 0 |
| hsa-miR-493-5p | 524 | 23.28 | 8577 | 385.07 | 16.54 | 0 |
| hsa-miR-154-5p | 1093 | 48.56 | 16617 | 746.03 | 15.36 | 0 |
| hsa-miR-142-3p | 643 | 28.57 | 9600 | 431.00 | 15.09 | 0 |
| hsa-miR-92b-3p | 259 | 11.51 | 3790 | 170.16 | 14.79 | 0 |
| hsa-miR-411-5p | 4889 | 217.22 | 67449 | 3028.18 | 13.94 | 0 |
| hsa-miR-485-5p | 173 | 7.69 | 2372 | 106.49 | 13.85 | 0 |
| hsa-miR-1268a | 61 | 2.71 | 809 | 36.32 | 13.40 | 1.38E-169 |
| hsa-miR-425-3p | 374 | 16.62 | 4905 | 220.21 | 13.25 | 0 |
| hsa-miR-455-3p | 813 | 36.12 | 10430 | 468.26 | 12.96 | 0 |
| hsa-miR-323a-3p | 120 | 5.33 | 1533 | 68.83 | 12.91 | 0 |
| hsa-miR-1268b | 71 | 3.15 | 853 | 38.30 | 12.14 | 3.80E-173 |
| hsa-miR-379-3p | 98 | 4.35 | 1139 | 51.14 | 11.74 | 5.03E-228 |
| hsa-miR-193a-5p | 539 | 23.95 | 6125 | 274.99 | 11.48 | 0 |
| hsa-miR-376c-3p | 4503 | 200.07 | 47659 | 2139.69 | 10.69 | 0 |
| hsa-miR-382-3p | 85 | 3.78 | 898 | 40.32 | 10.68 | 4.14E-174 |
| hsa-miR-223-3p | 1303 | 57.89 | 13355 | 599.58 | 10.36 | 0 |
| hsa-miR-21-5p | 194126 | 8625.22 | 1973835 | 88616.94 | 10.27 | 0 |
| hsa-miR-27a-5p | 99 | 4.40 | 956 | 42.92 | 9.76 | 6.73E-179 |
| hsa-miR-411-3p | 150 | 6.66 | 1396 | 62.67 | 9.40 | 1.79E-256 |
| hsa-miR-625-5p | 592 | 26.30 | 5433 | 243.92 | 9.27 | 0 |
| hsa-miR-487b-3p | 1197 | 53.18 | 10959 | 492.01 | 9.25 | 0 |
| hsa-miR-655-3p | 55 | 2.44 | 500 | 22.45 | 9.19 | 4.39E-92 |
| hsa-miR-337-3p | 851 | 37.81 | 7719 | 346.55 | 9.17 | 0 |
| hsa-miR-299-5p | 66 | 2.93 | 598 | 26.85 | 9.16 | 1.20E-109 |
| hsa-miR-181b-5p | 8565 | 380.55 | 76882 | 3451.68 | 9.07 | 0 |
| hsa-miR-656-3p | 141 | 6.26 | 1249 | 56.07 | 8.95 | 7.00E-225 |
| hsa-miR-342-3p | 6896 | 306.40 | 56032 | 2515.60 | 8.21 | 0 |
| hsa-miR-942-5p | 104 | 4.62 | 845 | 37.94 | 8.21 | 6.67E-147 |
| hsa-miR-100-5p | 27976 | 1243.00 | 222264 | 9978.72 | 8.03 | 0 |
| hsa-let-7i-5p | 77765 | 3455.18 | 600199 | 26946.43 | 7.80 | 0 |
| hsa-miR-1185-5p | 92 | 4.09 | 688 | 30.89 | 7.56 | 3.23E-115 |
| hsa-miR-654-3p | 431 | 19.15 | 2964 | 133.07 | 6.95 | 0 |
| hsa-miR-424-5p | 10353 | 459.99 | 70234 | 3153.21 | 6.85 | 0 |
| hsa-miR-941 | 254 | 11.29 | 1712 | 76.86 | 6.81 | 1.05E-268 |
| hsa-miR-125b-1-3p | 160 | 7.11 | 1032 | 46.33 | 6.52 | 1.00E-158 |
| hsa-miR-181a-5p | 67830 | 3013.76 | 435721 | 19562.05 | 6.49 | 0 |
| hsa-miR-410-3p | 121 | 5.38 | 743 | 33.36 | 6.20 | 1.65E-111 |
| hsa-miR-548k | 90 | 4.00 | 545 | 24.47 | 6.12 | 1.43E-81 |
| hsa-miR-10a-3p | 80 | 3.55 | 465 | 20.88 | 5.87 | 4.16E-68 |
| hsa-miR-671-5p | 118 | 5.24 | 651 | 29.23 | 5.57 | 1.62E-91 |
| hsa-miR-212-3p | 74 | 3.29 | 407 | 18.27 | 5.56 | 9.78E-58 |
| hsa-miR-26a-2-3p | 102 | 4.53 | 553 | 24.83 | 5.48 | 4.24E-77 |

Supplementary Table S3 43 downregulated differentially expressed known miRNAs in UPS tumor tissues compared with adjacent normal tissues

| miRNA ID | Copy number in  Adjacent normal tissue | Normalized miRNA level in Adjacent normal tissue | Copy number in UPS tissue | Normalized miRNA level in UPS tissue | Fold change | *P* value |
| --- | --- | --- | --- | --- | --- | --- |
| hsa-miR-133a-3p | 2579106 | 114592 | 438 | 20 | -5827.40 | 0 |
| hsa-miR-1-3p | 2398804 | 106581 | 440 | 20 | -5395.38 | 0 |
| hsa-miR-206 | 295147 | 13114 | 171 | 8 | -1708.13 | 0 |
| hsa-miR-499a-5p | 459811 | 20430 | 612 | 27 | -743.55 | 0 |
| hsa-miR-95-3p | 31853 | 1415 | 203 | 9 | -155.29 | 0 |
| hsa-miR-378a-5p | 13374 | 594 | 159 | 7 | -83.24 | 0 |
| hsa-miR-378d | 4958 | 220 | 62 | 3 | -79.14 | 0 |
| hsa-miR-378a-3p | 2171985 | 96504 | 30941 | 1389 | -69.47 | 0 |
| hsa-miR-486-5p | 8200 | 364 | 144 | 6 | -56.35 | 0 |
| hsa-miR-34a-5p | 24240 | 1077 | 492 | 22 | -48.76 | 0 |
| hsa-miR-378c | 34715 | 1542 | 1134 | 51 | -30.30 | 0 |
| hsa-miR-196a-5p | 7282 | 324 | 311 | 14 | -23.17 | 0 |
| hsa-miR-195-5p | 1375 | 61 | 66 | 3 | -20.62 | 1.94E-316 |
| hsa-miR-190b | 60649 | 2695 | 2970 | 133 | -20.21 | 0 |
| hsa-miR-193b-3p | 1244 | 55 | 61 | 3 | -20.18 | 3.93E-285 |
| hsa-miR-144-3p | 35535 | 1579 | 1830 | 82 | -19.22 | 0 |
| hsa-miR-30a-5p | 4997 | 222 | 268 | 12 | -18.45 | 0 |
| hsa-miR-1271-5p | 11236 | 499 | 682 | 31 | -16.30 | 0 |
| hsa-miR-497-5p | 338130 | 15023 | 24582 | 1104 | -13.61 | 0 |
| hsa-miR-24-3p | 33559 | 1491 | 2528 | 113 | -13.14 | 0 |
| hsa-miR-128-3p | 1120 | 50 | 93 | 4 | -11.92 | 2.53E-222 |
| hsa-miR-296-5p | 2787 | 124 | 233 | 10 | -11.84 | 0 |
| hsa-miR-486-3p | 4689 | 208 | 416 | 19 | -11.15 | 0 |
| hsa-miR-101-3p | 717668 | 31887 | 64312 | 2887 | -11.04 | 0 |
| hsa-miR-451a | 16626 | 739 | 1572 | 71 | -10.47 | 0 |
| hsa-miR-22-5p | 690675 | 30687 | 68380 | 3070 | -10.00 | 0 |
| hsa-miR-22-3p | 3479 | 155 | 361 | 16 | -9.54 | 0 |
| hsa-miR-1296-5p | 1111 | 49 | 118 | 5 | -9.32 | 5.51E-201 |
| hsa-miR-1303 | 67922 | 3018 | 7749 | 348 | -8.67 | 0 |
| hsa-miR-29c-3p | 1072 | 48 | 142 | 6 | -7.47 | 3.64E-175 |
| hsa-miR-152-3p | 82911 | 3684 | 12111 | 544 | -6.78 | 0 |
| hsa-miR-30d-5p | 1633 | 73 | 250 | 11 | -6.46 | 1.25E-245 |
| hsa-miR-4485-3p | 752 | 33 | 116 | 5 | -6.42 | 1.31E-113 |
| hsa-miR-675-3p | 517 | 23 | 81 | 4 | -6.32 | 6.15E-78 |
| hsa-miR-935 | 1400 | 62 | 226 | 10 | -6.13 | 2.78E-204 |
| hsa-miR-365a-3p | 1400 | 62 | 226 | 10 | -6.13 | 2.78E-204 |
| hsa-miR-365b-3p | 2275 | 101 | 377 | 17 | -5.97 | 0 |
| hsa-miR-181a-2-3p | 1802 | 80 | 332 | 15 | -5.37 | 1.16E-240 |
| hsa-miR-328-3p | 16952 | 753 | 3185 | 143 | -5.27 | 0 |
| hsa-miR-139-5p | 9670 | 430 | 1817 | 82 | -5.27 | 0 |
| hsa-miR-30e-5p | 733 | 33 | 141 | 6 | -5.14 | 4.88E-96 |
| hsa-miR-23b-5p | 1103 | 49 | 214 | 10 | -5.10 | 1.04E-142 |
| hsa-miR-99a-3p | 380 | 17 | 75 | 3 | -5.01 | 1.11E-49 |
